# Supplementary material for: BioPipelines: Accessible Computational Protein and Ligand Design for Chemical Biologists
Source: Comput Struct Biotechnol J. 2026 Jun 8;35(1):0129. doi: 10.34133/csbj.0129 (PMC13243794; doi:10.34133/csbj.0129)
Supplement: Supplementary 1 — Supplementary Text Figs. S1 to S13 Tables S1 to S4 Appendices A and B [file csbj.0129.f1.pdf]

Supplementary information for

BioPipelines: Accessible Computational Protein and Ligand Design for Chemical Biologists

Gianluca Quargnali*[1], Pablo Rivera-Fuentes*[1]

[1]Department of Chemistry, University of Zurich, 8056, Zurich, Switzerland.

*Correspondence to: gianluca.quargnali@chem.uzh.ch and pablo.riverafuentes@chem.uzh.ch

Supplementary figures

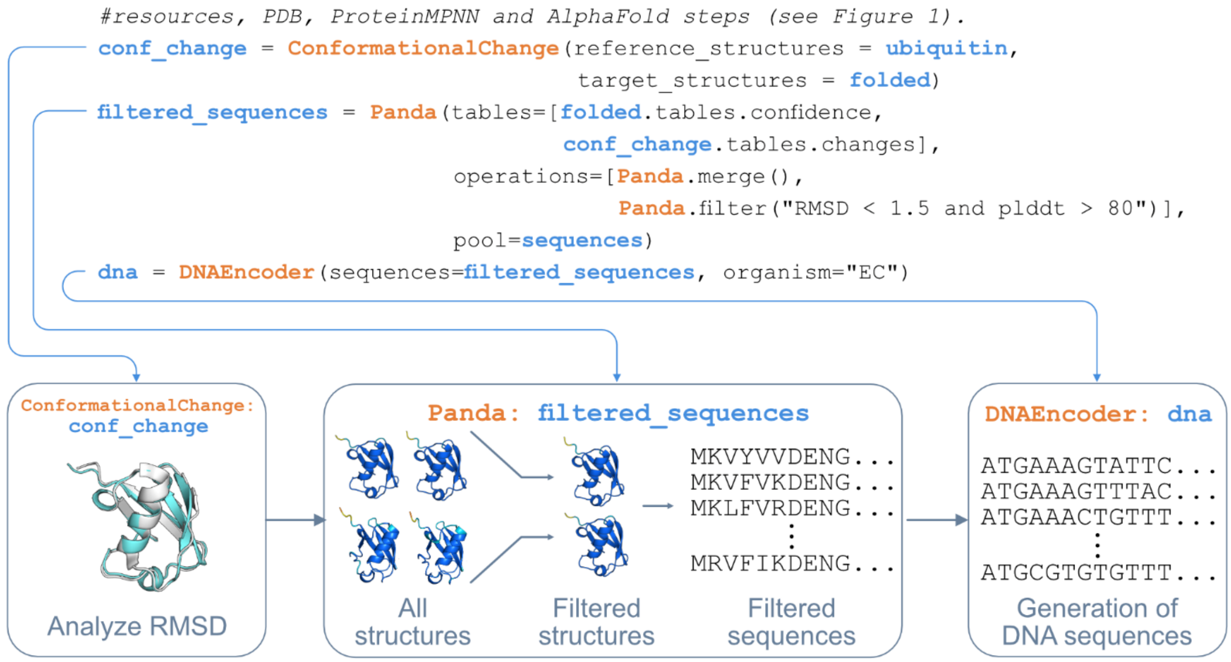

Figure S1. The tool ConformationalChange can be used to measure the RMSD between two sets of proteins matched by ID, like apo and holo Boltz folds, or, as done here, between a set of target proteins and a single reference structure. The output is a table containing per-structure RMSD data, which is here matched to the AlphaFold confidence by Panda. The resulting combined data is filtered to only include rows with RMSD < 1.5 Å and plddt > 80, and to extract pool elements, in this case protein sequences, matching the ids that pass the filter.

```
conf_change = ConformationalChange(reference_structures = kinase,
                                   target_structures = refolded,
                                   selection = backbones.tables.structures.fixed )
top3 = Panda(tables=[refolded.tables.confidence,
                     conf_change.tables.changes],
             operations=[Panda.merge(),
                         Panda.filter("RMSD < 1.5")],
                         Panda.sort("plddt",
                                    ascending = False)],
                         Panda.head(3)],
             pool=refolded)
PyMOL(PyMOL.Load(top3),
      PyMOL.ColorAF(),
      PyMOL.Color("white",selection=backbones.tables.structures.fixed)
```

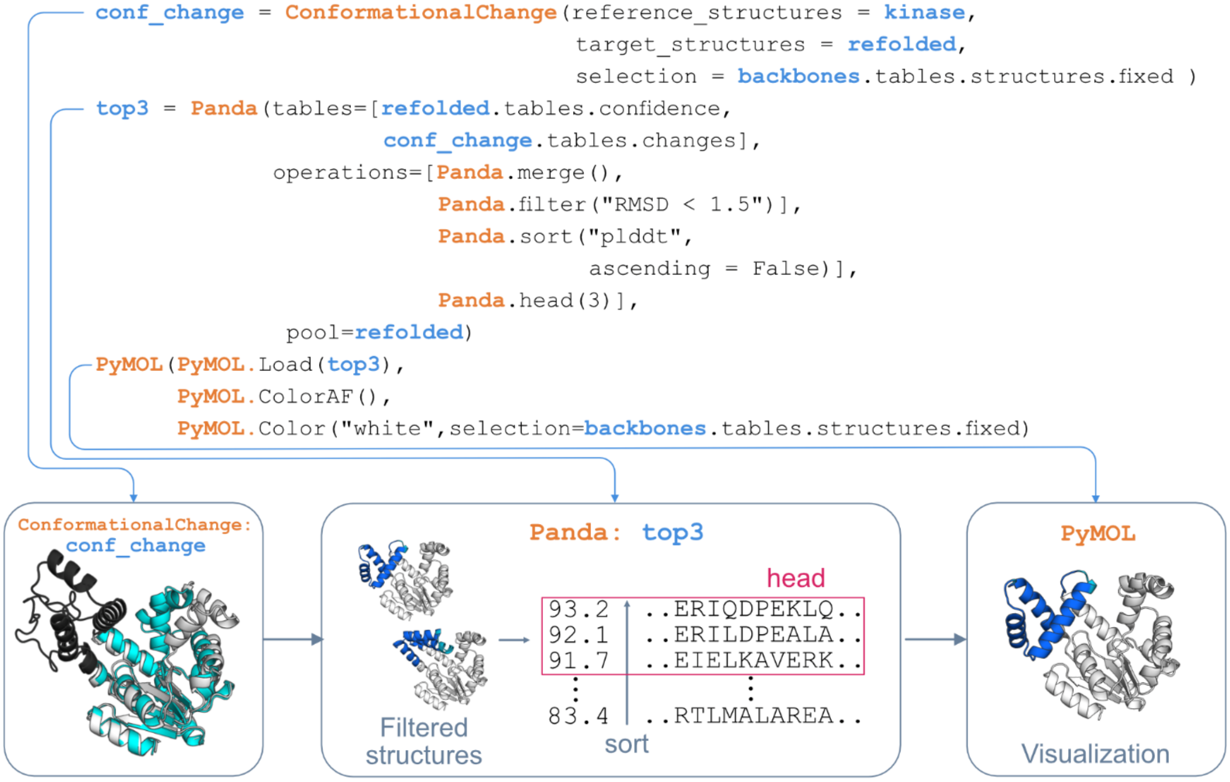

Figure S2. Structures whose id pass the series of pandas operations are transferred from the pool to top3. A PyMOL session is created, already displaying the portions of interest.

```
with Pipeline(project="TrpR", job="LibraryScreen"):
    # Resources, Sequence, and Boltz2 identical to Figure3
    SMILES_library = {"candidate": r"<R1>CC1=CNC2=CC=CC=C21",
                      "R1": ["<amine>", "<amino acid>", "<acid>"],
                      "amine": r"[C@@H](N)(C(C)(C)C)",
                      "amino acid": r"[C@@H](N)(C(=O)O)",
                      "acid": r"[C@@H](C(C)(C)C)(C(=O)O)"}
    library = CompoundLibrary(SMILES_library,
                              generate_images = True)
```

CompoundLibrary: library

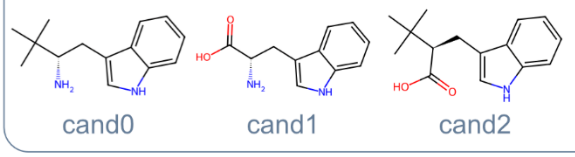

id	format	smiles	ccd	R1
cand0	smiles	[C@@H](N)(C(C)(C)C)...		amine
cand1	smiles	[C@@H](N)(C(=O)O)C...		amino acid
cand2	smiles	[C@@H](C(C)(C)C)(C(=...		acid

Figure S3. A compound library can be defined, besides importing a ChemDraw file with R-group tables, also with a SMILES dictionary (which can be loaded from a csv file). We recommend generating compound images for inspection.

```
merged = Panda(tables=[cofolded.tables.affinity,
                       library.tables.compounds],
               operations=[Panda.merge(),
                           Panda.calculate({"aff_uM":"10**affinity_pred_value"})])
Plot(Plot.Scatter(data=merged.tables.result,
                  title="Predicted Affinity by R1",
                  x="R1",
                  y="aff_uM",
                  xlabel="R1 group"
                  ylabel="Predicted Affinity [uM]"))
```

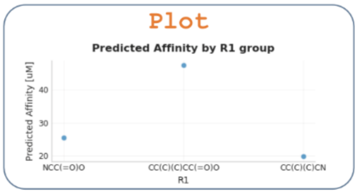

Figure S4. The predicted affinity is converted into micromolar units with Panda and plotted against the R1 substituent. When names are not provided for each R1 group in the ChemDraw XML file, the SMILES corresponding to each fragment are displayed.

```python
mutants = Mutagenesis(original=fusions,
                      position=Selection(Selection.add(fusions.tables.sequences.L1),
                                         Selection.add(fusions.tables.sequences.L2))
                      mutate_to="LK",
                      combinatorial=True)
# Following steps using sequences from mutants instead of fusions
```

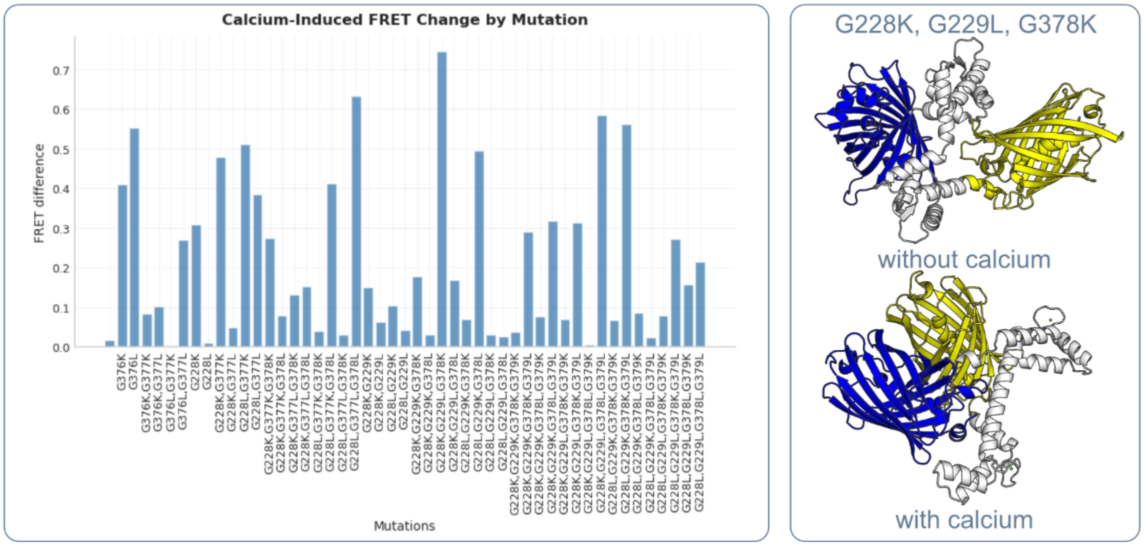

Figure S5. Combining linker length and residue screening when designing a FRET sensor. After fusing the sequences with the Fuse tool, here we mutate all the linker positions, which are defined in the sequences table of the Fuse output, populated at runtime, using the Mutagenesis tool. We combine the two residue selections with the Selection tool and feed all the positions to a single instance of Mutagenesis. We chose to mutate to either leucine (L) or lysine (K). By setting the combinatorial mode, we make sure all the resulting sequences have all the residues defined in the selections mutated at once.

```
with Pipeline(project="FRET", job="FRET sensor"):
    dist_apo = Distance(structures = apo,
                        residue = ["66","-173"],
                        metric_name = "FRET_distance_apo")
    dist_holo = Distance(structures = holo,
                        residue = ["66","-173"],
                        metric_name = "FRET_distance_holo")
    R0 = 35.4 # Angstrom
    derived_metrics = {"FRET_E_apo": f"1 / (1 + (FRET_distance_apo / {R0})**6)",
                      "FRET_E_holo": f"1 / (1 + (FRET_distance_holo / {R0})**6)",
                      "delta_FRET": "abs(FRET_E_holo - FRET_E_apo"}
    analysis = Panda(tables=[fusion.tables.sequences,
                             dist_apo.tables.distances,
                             dist_holo.tables.distances],
                     operations=[Panda.merge(),
                                 Panda.calculate(derived_metrics)])
    Plot(Plot.Bar(data=analysis.tables.result,
                  title="FRET efficiency by linker length",
                  x="lengths",
                  y="FRET_E_apo",
                  y_right="FRET_E_holo",
                  xlabel="Linker Lengths"
                  ylabel="FRET apo",
                  ylabel_right_right="FRET holo"))
```

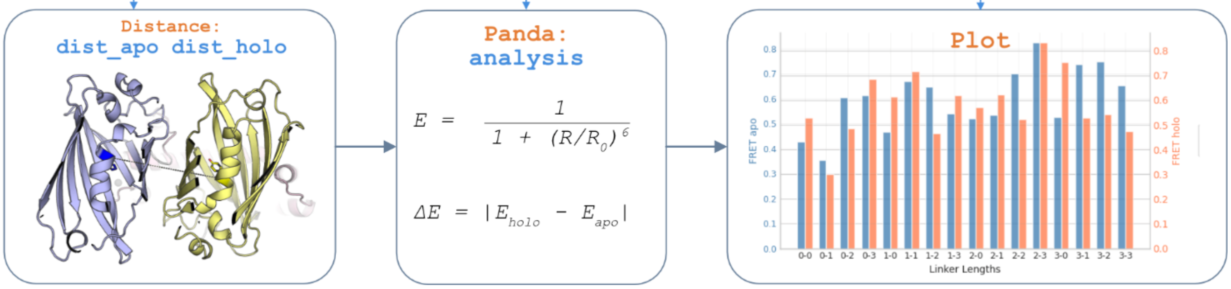

Figure S6. Modelling a FRET-based calcium sensor based on calmodulin and two fluorescent proteins. With the Distance tool, we measure the distance between the two fluorophores, positioned at residue 66 in EBFP, and -173 (counting from the C terminus) in EYFP. Further, we use Panda to calculate derived metrics based on the Förster radius of 35.4 Å for the EBFP-EYFP couple, and plot the results against the linker lengths.

```python
with Pipeline(project="Kinase", job="LID_Redesign_Inspection"):
      kinase = PDB("4AKE")
      backbones = RFdiffusion(pdb=kinase),
                              contigs='A1-117/50-70/A161-214',
                              num_designs=10)
      sequences = ProteinMPNN(structures=backbones,
                              num_sequences=2,
                              redesigned=backbones.tables.structures.designed)
sequences
```

sequences

STREAMS

name	format	items	map_table
sequences	csv	20	<output_folder>/queries.csv

sequences

name	format	items	map_table	files_contain_wildcards
sequences	csv	20	<output_folder>/queries.csv	False

id	gaps	sequence	T	sample	score	global_score	seq_recovery	structures.id
LID_Redesign_002_6_1		MRIILLGAPGAGKGTQAQFIMEKYGIPQISTGDMLRAAVKSGSELGKQAKDIMDAGK...	0.1	1	1.0641	1.477	0.0299	LID_Redesign_002_6
LID_Redesign_002_6_2		MRIILLGAPGAGKGTQAQFIMEKYGIPQISTGDMLRAAVKSGSELGKQAKDIMDAGK...	0.1	2	1.0837	1.4531	0.0299	LID_Redesign_002_6
		... 16 more ...						
LID_Redesign_002_3_1		MRIILLGAPGAGKGTQAQFIMEKYGIPQISTGDMLRAAVKSGSELGKQAKDIMDAGK...	0.1	1	1.1609	1.5006	0.02	LID_Redesign_002_3
LID_Redesign_002_3_2		MRIILLGAPGAGKGTQAQFIMEKYGIPQISTGDMLRAAVKSGSELGKQAKDIMDAGK...	0.1	2	1.1604	1.5023	0.04	LID_Redesign_002_3

TABLES

name	columns	path
sequences	id, structures.id, source_pdb, sequence, score, seq_recovery, gaps	<output_folder>/queries.csv
missing	id, removed_by, cause	<output_folder>/missing.csv

```python
with Pipeline(project="AdenylateKinase", job="LID_Redesign_Inspection"):
      backbones = Load("/path/to/002_RFdiffusion")
backbones
```

backbones

STREAMS

name	format	items	map_table
structures	pdb	10	<output_folder>/structures_map.csv

structures

name	format	items	map_table	files_contain_wildcards
structures	pdb	10	<output_folder>/structures_map.csv	False

id	file	value
LID_Redesign_002_1	/shares/locbp.chem.uzh/gquarg/BioPipelines/AdenylateKinas...	
LID_Redesign_002_2	/shares/locbp.chem.uzh/gquarg/BioPipelines/AdenylateKinas...	
	... 6 more ...	
LID_Redesign_002_9	/shares/locbp.chem.uzh/gquarg/BioPipelines/AdenylateKinas...	
LID_Redesign_002_10	/shares/locbp.chem.uzh/gquarg/BioPipelines/AdenylateKinas...	

TABLES

name	columns	path
structures	id, source_id, pdb, fixed, designed, contigs, time, status	<output_folder>/rfdiffusion_results.csv

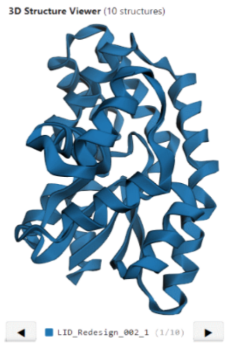

Figure S7. Top: Inspection of an intermediate tool's output of a pipeline run in Jupyter. Bottom: Inspection of a tool's output from a previously run pipeline.

Supplementary figures

Table S1. Tools currently implemented in BioPipelines.

Function	#	Tool	Description	Ref.
Structure generation	1	RFdiffusion	Protein backbone generation	[1]
	2	RFdiffusionAllAtom	Backbone generation, atoms-aware	[2]
	3	RFdiffusion3	Protein generation	[3]
	4	BoltzGen	Protein generation and analysis	[4]
Sequence design	5	ProteinMPNN	Inverse folding	[5]
	6	LigandMPNN	Inverse folding, ligand-aware	[6]
	7	Mutagenesis	Site-directed mutagenesis	
	8	MutationComposer	Sampling-based sequence generation	
	9	Fuse	Combinatorial fusing of sequences	
	10	StitchSequences	Stitching of sequences	
	11	DNAEncoder	Inverse-translation	[7]
	12	RBSDesigner	Optimization of the ribosomal binding site	[8]
Structure prediction	13	AlphaFold2	Protein folding	[9]
	14	Boltz2	Folding of several molecular types	[10]
	15	Gnina	Molecular docking with deep learning	[11]
Basic input types	16	Ligand	Ligand loading incl. PubChem, RCSB	
	17	PDB	Protein complexes loading incl. RCSB	
	18	RCSB	Data loading from RCSB using Query API	
	19	CompoundLibrary	SMILES library expansion	
	20	Sequence	Protein, DNA, RNA sequences loading	
	21	Table	Table loading from files	
Analysis	22	Distance	Distance calculations (atoms, residues)	
	23	Angle	Angle and dihedral calculations	
	24	DistanceSelector	Proximity-based residue selection	
	25	Contacts	Contacts between ligands and proteins	
	26	ConformationalChange	Measurement of structural changes	
	27	PoseChange	Measurement of ligand pose changes	
	28	PoseBusters	Conformational plausibility	[12]
	29	SASA	Solvent-accessible surface area on binding	
	30	ADMETAI	ADMET endpoint predictions for compounds	[15]
	31	CABSflex	Coarse-Grained Monte Carlo	[13]
Sequence statistics	32	MutationProfiler	Profiling of amino acid frequencies	
	33	SequenceMetricCorrelation	Correlation between a sequence and a metric	
	34	BayesianAdjuster	Frequency adjustment based on correlations	
MSAs	35	MMseqs2, MMseqs2Server	Multiple sequence alignment (uniref30, GPU)	[14]

	36	MSA	MSA format conversion (CSV/A3M)	
Table operations	37	Panda	Table operations and stream filtering	
	38	ExtractMetrics	Metric extraction from several tables	
	39	ReMap	Rename IDs across streams and tables	
	40	Selection	Selection-string manipulation	
Visualization	41	PyMOL	Sessions with aligned and colored structures	
	42	Plot	Various kinds of plots	
Utility	43	Load, LoadMultiple	Loading of previously run tools	
	44	Pool	Aggregate parallel-run outputs into one stream	

Table S2. Parameters not covered within the current implementation of the tools.

Tool	Deferred categories (one example flag each)
ProteinMPNN	PSSM-driven sampling (--pssm_jsonl); per-residue biasing (--bias_by_res_jsonl); tied / chain-wise positions (--tied_positions_jsonl); scoring- and probability-only modes (--score_only); Cα-only model variant (--ca_only); global AA omission (--omit_AAs)
LigandMPNN	per-residue omission (--omit_AA_per_residue); symmetry / homo-oligomer design (--symmetry_residues); ligand-context toggles (--ligand_mpnn_use_side_chain_context); transmembrane modes (--transmembrane_buried); scoring-only modes (--score_only); side-chain packing (--pack_side_chains)
RFdiffusion	symmetric / cyclic backbones (inference.symmetry); PPI hotspots (ppi.hotspot_res — exposed in AllAtom); secondary-structure-aware inpainting (contigmap.inpaint_str_helix); scaffold-guided generation (scaffoldguided.*); guiding potentials (potentials.guiding_potentials); low-level diffuser/denoiser schedule knobs (denoiser.noise_scale_ca); trajectory dumping (inference.write_trajectory)
RFdiffusionAllAtom	symmetric backbones (inference.symmetry); low-level denoiser-noise / ligand-conformer schedule knobs
RFdiffusion3	none — every foundry-config key reachable via the json_config escape hatch
AlphaFold (ColabFold)	template-conditioned folding (--templates); MSA strategy (--msa-mode); model selection / ensembling (--num-models); AMBER-relax tuning (--relax-tolerance); secondary outputs (--save-all); advanced inference (--use-dropout)
Boltz-2	non-canonical / modified residues inside a chain via CCD codes (`modifications`, e.g. pSer, MSE, distinct from `glycosylation` and `covalent_linkage`, both exposed); cyclic chains; sampler tuning (--sampling_steps); MSA-server authentication (--api_key_value); affinity-prediction tuning (--affinity_mw_correction); MSA subsampling (--subsample_msa); secondary outputs (--write_full_pae)
BoltzGen	external checkpoints (--folding_checkpoint); inverse-folding-only / avoidance modes (--only_inverse_fold); design-size bucketing (--

	size_buckets); model-cache and downloader settings (--moldir); execution-mode toggles (--no_subprocess)
Gnina	flexible side-chain docking (`--flex`); covalent docking (`--covalent_rec_atom`); Monte-Carlo sampler tuning (`--num_mc_steps`); minimisation-only modes (`--minimize`, `--randomize_only`); custom scoring functions (`--custom_scoring`); CNN model selection and weight mixing (`--cnn_model`, `--cnn_mix_emp_force`); scoring- and local-only modes (`--score_only`, `--local_only`); ligand preprocessing toggles (`--addH`, `--stripH`); pose-output handling (`--pose_sort_order`, `--atom_terms`)
MMseqs2	functionalities not related to protein folding (many-against-many)
CABSflex	--seed; --simulation_type
PyMOL	raw cmd.X API

Architecture

1. **Multimodal execution with a simple syntax**

BioPipelines was developed around a desired clear, simple syntax according to which a tool is configured and run by simply writing its name together with its own parameters, and the output can be passed to subsequent tools without acting on it. This result was achieved through a combination of architectural choices and Python's programming language innate features. Namely, a BioPipelines workflow is defined within a Python context manager *with*, through a general Pipeline class, such that tools instantiated inside the context are automatically registered with the pipeline. The autoregistration mechanism is implemented once and for all in the __*new*__ method, as defined in the abstract class BaseConfig, from which all tool configurators inherit. The Pipeline object is responsible for coordinating the tools, that is, chaining their execution, and defining the broad filesystem structure. In general, each pipeline produces a self-contained directory (**Figure S8**). During the configuration phase, the framework predicts all output file paths and generates self-contained bash execution scripts, which are stored in the runtime directory for potential inspection. Each tool's output resides in a numbered, tool-named subdirectory containing the expected output as well as its execution log.

```
<biopipelines_output>/<project>/<job>_<NNN>/
├── RunTime/                    # Execution scripts
│   ├── pipeline.sh
│   ├── 001_<tool>.sh
│   └── 002_<tool>.sh
├── Logs/                       # Execution logs
│   ├── 001_<tool>.log
│   └── 002_<tool>.log
├── ToolOutputs/                # Tool output predictions
│   ├── 001_<tool>.json
│   └── 002_<tool>.json
├── 001_<Tool>/                 # Tool outputs
│   ├── .expected_outputs.json
│   ├── .log
│   └── various output (PDBs, CSVs, ...)
└── 002_<Tool>/
...  └── ...
```

Figure S8. Self-contained output organization of a pipeline's output.

The context manager allows for the pipeline to pick up not only tool configurations as they are added, but also intermediate directives for the overall pipeline script being generated. For example, resources, dependencies, or simple folder name manipulation with a suffix can be appended in the same syntax and are applied either to the slurm script or to the file system structure. A dedicated submission script accessible after package installation via command line interface (CLI) *bp-submit <pipeline.py>* collects the coordinator scripts generated during the configuration and submits them while incorporating potential intra-pipeline dependencies, thus allowing the user to define multi-job pipelines within the same framework (**Figure S9**). Furthermore, with its simple on-the-fly parameter, automatically set to True when a notebook is detected, each tool script *<N>_<tool>.sh* is run as soon as it is generated.

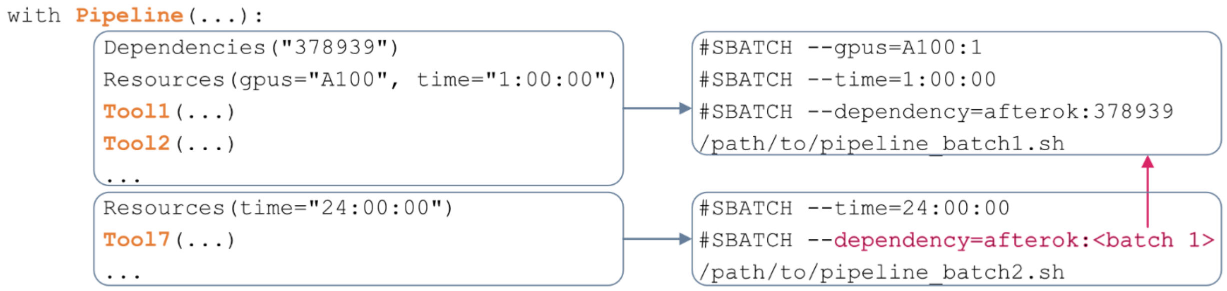

Figure S9.Keywords *Dependencies* and *Resources* are interpreted by the biopipelines submit script. When requesting two (or more) resources within the same pipeline, the script will generate and submit two (or more) SLURM batches, each one dependent on the previous one.

2. Standardized interfacing of tools

There are three main challenges in chaining tools within a pipeline: (i) tools organize their outputs arbitrarily; (ii) the parenting relationship between input and output data is possibly lost during execution; and (iii) tools produce and consume different data types for same entity types, for example, common PDB or CIF for structures, CSV or fasta for sequences, and so on. BioPipelines addresses these challenges with the introduction of a StandardizedOutput class exposing a collection of any of two complementary data containers: streams and tables (**Figure S10**).

```
@dataclass
class Datastream:
        conf_change = str            # "structures", "sequences", ...
        ids = List[str]              # Unique identifiers in pattern form
        map_table = str              # CSV path mapping IDs to files, values, metadata
        files = List[str]            # File paths, in pattern form
        format = str                 # "pdb", "cif", ...
        metadata = List[str, Any]    # Additional stream information


@dataclass
class TableInfo:
        name= str                    # "confidence", "affinity", ...
        path = str                   # Path to CSV file
        columns = List[str]          # Expected column names
        description = str            # Human-readable description
```

Figure S10. Public attributes of the DataStream and TableInfo class.

Streams (DataStream) represent ID-tracked collections of entities that are produced by and flow between tools. In particular, each stream associates unique identifiers with either files (*.pdb*, *.cif*, …) or values (*"MHHHHHHLTKW…", "ATP", …*), and records the data format. This source tool-agnostic definition of the output implies that, for example, ProteinMPNN can operate on the structures produced by RFdiffusion, PDB, or AlphaFold, without distinction, because all it receives is a DataStream identically formatted.

Stream objects are constructed deterministically during the configuration phase by the tool configurator classes, based on input streams from previous tools and tool-specific parameters. This happens as soon as the tools are added to the pipeline, before the files or values exist. The parent-tool tracking, necessary to compare or combine data from different tools, happens by following a series of simple conventions for ID generation, based on a tool's input vs output relationship:

- 1 entity → 1 entity: output IDs are identical to input IDs. *Example: AlphaFold: 1 sequence → 1 best-ranked model*
- 1 entity → N entities: output IDs are obtained by appending an underscore and a suffix, determined by tool-specific parameters. *Example: ProteinMPNN: 1 structure → N sequences where N is num_sequences. 3KU8 → 3KU8_<1..N>.*
- J entities x K entities x … → N entities: output IDs are combinations of input ones, separated by a + character, and map_table will contain the source IDs as provenance. *Example: Boltz2: [prot1, prot2, …] x [lig1,lig2,…] → [prot1+lig1,prot1+lig2,…].*

The Biopipelines Python package provides standardized functions to handle streams and to match data derived by upstream or downstream tools based on these conventions, and the ReMap tool for reassigning IDs, for example, to combine outputs coming from different pipelines.

Tables (TableInfo) represent tabular metadata associated with streams. They describe CSV files and predict column names as well. Tables always present a first column called *id* for matching data to streams, but they are not ID-tracked to allow easier operations, with the tool Panda performing both table operations and filtering of streams based on table data.

When a tool registers itself within an active pipeline context, it returns a *StandardizedOutput* object rather than the tool instance itself. This design is consistent with the principle that once a tool's configuration is concluded, i.e., when the constructor arguments are set, only the predicted output is needed by downstream tools. Further, the *StandardizedOutput* is serializable, such that each tool saves its own predicted output in its output folder as a JSON file, and the file itself can be loaded with the Load tool to obtain an object that behaves identically to the original input. This serialization enables workflows to be resumed or extended across sections.

Finally, we address the problem of different formats with some basic input entity types that ensure representation coherence across the framework. These types are strictly associated with standard streams. The general practice is that whenever a tool accepts, for example, a protein sequence, it does so by receiving the output of a Sequence object instantiated with the given sequence (**Figure S11**). By enforcing this behavior, tools are guaranteed to operate on tool output streams in the same way as they operate on simple inputs. To extend the framework to new basic types, all that is there to be done is to define a new stream name. The dot access notation holds for any stream name.

```
with Pipeline(...):
        # correct: receives sequences stream from Sequence as from any other tool
        Tool(proteins=Sequence("MHHHHHHGTRK..."))
        # wrong: non-standardized input, no id tracking
        Tool(proteins="MHHHHHHGTRK...))
```

Figure S11. Convention for passing stream types as arguments to tool constructors.

Tool integration

All tools inherit from BaseConfig. Conformance to the framework requires implementing four methods (**Figure S12**): validate_params, which checks the parameters supplied to the constructor; configure_inputs, which receives the pipeline's folder dictionary once the tool has been added to a pipeline; generate_script, which emits the bash script that executes the tool; and get_output_files, which declares the files the tool is expected to produce so that the framework can verify completion and expose the outputs as standardized streams. The base class also provides working defaults for configuration display (get_config_display), which is by default injected into the log output; in practice, fully integrated tools override these to surface their tool-specific parameters in notebook output and persisted records. Beyond the interface itself, a companion parser in HelpScripts/pipe_*.py that converts the tool's native outputs into the standardized stream representation, and updates file maps based on the runtime results, including the generation of a “missing” table with cause, if required. The implementation of _install_script and addition of environment specifications is an optional add-on since tools can be installed independently, as long as the right environment name is configured in the config file.

```
class BaseConfig(ABC):
        TOOL_NAME: str
        def validate_params(self):
        # checks parameters are correct, e.g., num_sequences > 0
        def generate_script(self):
        # generates a bash script for tool execution
        def get_output_files(self):
        # predicts DataStream and TableInfo outputs
        def configure_inputs(self, pipeline_folders: Dict[str,str]):
        # receives folders structure from the active pipeline
        def get_config_display(self):
        # provides a text paragraph with the configuration specifications
        def _install_script(self,folders, env_manager, force_reinstall, **kwargs):
        # produces a script to install the tool

class ProteinMPNN(BaseConfig):
        ...
        def generate_script(self):
         """Generate ProteinMPNN execution script."""
         script_content = "#!/bin/bash\n"
         script_content += "# ProteinMPNN execution script\n"
         script_content += self.generate_completion_check_header()
         script_content += self.activate_environment()
         script_content += self._generate_script_prepare_inputs()
         script_content += self._generate_script_run_proteinmpnn()
         script_content += self._generate_script_create_table()
         script_content += self.generate_completion_check_footer()
         return script_content
```

Figure S12. Base configurator class and example of script generation.

Each generated bash script follows a standard five-step structure:

1. Completion check: Skip execution if a completion marker already exists.
2. Environment activation: Activate the tool's conda environment as specified in config.yaml.
3. Tool execution: Run the tool's command-line interface with the configured parameters.
4. Output parsing: Execute a helper script (pipe_*.py from HelpScripts/) to parse tool-specific outputs into standardized CSV tables.
5. Output verification: Check that all expected outputs exist and create a completion marker on success.

Steps (1) and (5) are handled by biopipelines via a shared script since every tool's own output is defined as a JSON-serialized object, and also environment activation based on machine configuration is handled by the package. An interrupted pipeline can be reubmitted with the *bp-resubmit /path/to/slurm.sh* command, and every tool whose marker already exists will be skipped.

Configuration

The configuration files at the repository root specify machine-specific settings: output paths, conda environments for each tool, scheduler, and account information (**Figure S13**). As a default, BioPipelines employs the config.colab.yaml when Google Colab is detected, whereas other variants are activated by matching the declared username against the system's. Environment definitions are also variant-keyed, so a single repository can carry, e.g., a cluster variant (SLURM + mamba + apptainer) and a colab variant (no scheduler + micromamba + no container) side by side.

Site-specific adaptation is handled through a dedicated CLI, bp-config, with two interactive subcommands. bp-config auto probes the host, detecting the username, the environment manager on the PATH, the scheduler (by querying sinfo), the available modules and Lmod profile, the container runtime (apptainer or singularity), and the user's git e-mail (for job updates), and writes the discovered values into the chosen variant. bp-config edit opens any variant in a text-based interactive editor, highlighting folders that do not resolve on the host.

```yaml
machine:
  username: your_username
  emails:
    your_username: "your.email@institution.edu"
  env_manager:
    name: "mamba"
    init:
    - 'eval "$(mamba shell hook --shell bash)"'
  scheduler:
    name: "slurm"
    init:
    - 'for f in /etc/profile.d/lmod.sh /etc/profile.d/z*_lmodenv.sh; do [ -f "$f"
      ] && source "$f"; done'
    modules:
    - "miniforge3"
    - "apptainer"
  container_executor: "apptainer"

folders:
  base:
    home: "/home/<username>"
    data: "<home>/data"                    # where tool repositories are installed
    group: "/shares/your_group"            # shared group storage
    user: "<group>/<username>"
    biopipelines: "<cwd>"                  # auto-set to repository root
    biopipelines_output: "<user>/BioPipelines"

  infrastructure:
    pipe_scripts: "<biopipelines>/pipe_scripts"
    pdbs: "<biopipelines>/pdbs"
    ligands: "<biopipelines>/ligands"
    sequences: "<biopipelines>/sequences"
    tables: "<biopipelines>/tables"
    cache: "<group>/cache"
    containers: "<group>/containers"

  # Tool installation paths
  repositories:
    RFdiffusion: "<data>/RFdiffusion"
    ProteinMPNN: "<data>/ProteinMPNN"
    LigandMPNN: "<data>/LigandMPNN"
    # ...

environments:
  RFdiffusion: "SE3nv"
  ProteinMPNN: "SE3nv"
  LigandMPNN: "ligandmpnn_env"
  # ...
```

Figure S13. Machine configuration via yaml file.

Reproducibility

Reproducibility artifacts are captured per run via Pipeline(debug=True), which writes a _debug_capture/ directory alongside the outputs. At configuration time it records the active config.yaml, the BioPipelines framework version, and the per-tool wrapper ersion, environment name, and container image in tools.json. At execution time the generated bash script appends mamba env export / pip freeze per environment plus uname, nvidia-smi, and scheduler/container-executor versions, so the runtime state of the executing node is recorded next to the outputs. BioPipelines does not enforce upstream tool versions; reproducibility is achieved by recording

resolved versions rather than pinning them up front, and model weights are identified by the resolved model name in the per-tool config.

Comparison with existing frameworks

We compare BioPipelines with the available software most relevant to protein-design workflows: ProtFlow, Ovo, ProteinDJ, ColabFold, and the workflow engines Nextflow and Snakemake. The last two are general-purpose frameworks for defining HPC-runnable workflows; since they provide no protein-design-specific functionality, we treat them as representatives of manual scripting. **Table S3** compares them on relevant axes: scope, architecture, supported platforms, reproducibility, and extensibility.

What most distinguishes BioPipelines is how typed data streams and the associated ID system shape both development and use: a tool wrapper is written against a stream type, not against the tool that produced it. Adding a tool is therefore a self-contained operation, with no edits to any other tool or to the framework core, and the new tool is immediately usable upstream or downstream of every existing one. The ID system carries this through execution, without the user maintaining filename conventions. ProtFlow comes closest to this overall: it wraps a comparable range of design, folding, docking, and MD tools and tracks the same lineage, but its tools exchange data through prefix-namespaced columns on a single shared DataFrame that the user threads by hand, so both interoperability and lineage rest on a naming convention rather than a typed contract. The other systems are more constrained by construction: ProteinDJ and ColabFold are fixed pipelines rather than general toolkits, so adding a tool is not a user-level operation but a change to the pipeline itself, and lineage is tracked only through their own metadata keys; Ovo is extensible, but through a heavier unit (pip-installable plugin), making extension a developer task rather than something a typical user can do; and Nextflow and Snakemake will run any tool only because the user writes the wrapper, the file conversions, the cross-tool joins, and any lineage tracking from scratch each time.

Table S3. Feature comparison

Feature	BioPipelines	ProtFlow	ColabFold	Ovo	ProteinDJ	Nextflow / Snakemake
**Scope**	protein and sequence design + (co)folding + docking + scoring	protein and sequence design + folding + docking + MD + scoring	AlphaFold-family structure-prediction inference	protein design + sequence design + validation	de novo binder design pipeline (fixed recipes)	general directed acyclic graph (DAG) engines
**Typing; Lineage**	yes — typed DataStream / StandardizedOutput ; per-ID provenance and suffix conventions	no — single Poses pandas DataFrame; lineage carried by prefix convention threaded by the user	n/a — single-tool inference	no — relational DB of design records + descriptors + jobs; lineage via design→job links and per-run-prefixed IDs.	no — Nextflow topic channels (PDB + paired JSON); lineage = fold_id / seq_id keys	no — untyped Nextflow channels / Snakemake file paths; no built-in cross-tool ID matching
**Data model**	per-run filesystem; table CSVs per tool on disk, joined lazily	per-run filesystem; one global in-memory poses.df	per-run filesystem; single inference output table	persistent SQL database	per-run filesystem; streaming channels + on-disk PDB/JSON pairs + final CSV	per-run filesystem; intermediate files streamed between processes
**Colab / HPC**	✓ / ✓ (SLURM)	✓ / ✓ (SLURM)	✓ / ✓ (LocalColabFold)	— (web UI) / ✓ (Nextflow-based) — web UI	— / ✓ (SLURM + Seqera)	— / ✓ (SLURM, SGE, LSF, …)
**Container support**	apptainer / singularity, per tool	no	docker image available	docker / singularity	apptainer (required)	docker / singularity / apptainer, first-class
**Reproducibility capture**	debug mode: config snapshot, tool + framework versions, env exports, machine data	manual (logs + cmd-files)	n/a	DB provenance records + Nextflow run metadata	configs + inputs copied out, nextflow.log, trace files, pinned model checkpoints	Nextflow: -with-report / -with-trace / -with-timeline; Snakemake: conda-env capture + interactive HTML reports
**Extensibility**	subclass BaseConfig + add a pipe_<tool>.py for output standardization	subclass the Runner class; add the tool path to config.py	n/a	write a pip plugin: a Streamlit UI page + a DesignWorkflow subclass + Descriptor types + a Nextflow process	add a Nextflow process following the PDB + JSON + metadata convention + matching Apptainer image	write a new Nextflow DSL2 process module / Snakemake rule

Framework performance and scalability

We measured the wall-clock time of Pipeline.save() as the minimum of three runs on a S3IT login node, along two axes: a fixed three-tool pipeline:

AlphaFold(ProteinMPNN(RFdiffusion(num_designs=N, contigs="100-150")))

with the design count N scaled from 10 (18 ms) to 10000 (26 ms), and a ProteinMPNN↔AlphaFold ping-pong off a PDB("4AKE") input with the chain length scaled from 10 (0.674 s) to 1000 (7.906 s) total tools. Thus, Pipeline.save() operation completes within seconds for any reasonable pipeline.

We measured likewise the recurring runtime cost of cross-tool ID matching for a parent→child merge of N source against N target identifiers via id_map_utils.get_mapped_ids, via suffix stripping (0.67 µs/id, 6.87 ms at N=10000) and provenance-CSV (7.66 µs/id, 78.72 ms at N=10000). Even though ID-matching can happen many times within a pipeline, i.e. a few times per tool, th runtime overhead introduced is still negligible relative to the computational cost of typical protein design workflows. For comparison, a single RFdiffusion design typically requires 1 minute of GPU time, placing the framework overhead below 10^{-4} of total wall-clock time.

Scalability is enabled by number of entities-independent execution script size, with larger campaigns differing only in the number of rows in the mapping tables, and by using algorithms for runtime ID matching that are O(n). In addition, BioPipelines stores intermediate outputs as per-tool CSV files and performs lazy joins, such that peak memory usage is bounded by the largest individual tool output rather than the cumulative size of all intermediate data. This design extends the practical scale of campaigns where a single monolithic table would otherwise exhaust available memory.

Table S4. Runtime overhead and scalability.

**Metric**	**Result**	**Interpretation**
Pipeline.save() runtime (10 to 10k IDs)	Independent of N	IDs not materialized yet
Pipeline.save() runtime (10 to 1k tools)	< 10 seconds	Negligible for common tasks
Merge overhead (10k IDs, suffix)	45 ms	Negligible
Merge overhead at (10k IDs, provenence)	460 ms	Negligible
Typical RFdiffusion runtime (A100 GPU)	1 min/design	Dominates total runtime
Peak memory scaling	Bounded by N	Avoids cumulative table

References

[1] J. L. Watson, D. Juergens, N. R. Bennett, B. L. Trippe, J. Yim, H. E. Eisenach, W. Ahern, A. J. Borst, R. J. Ragotte, L. F. Milles, B. I. M. Wicky, N. Hanikel, S. J. Pellock, A. Courbet, W. Sheffler, J. Wang, P. Venkatesh, I. Sappington, S. V. Torres, A. Lauko, V. De Bortoli, E. Mathieu, S. Ovchinnikov, R. Barzilay, T. S. Jaakkola, F. DiMaio, M. Baek, D. Baker, "De novo design of protein structure and function with RFdiffusion" *Nature* **2023**, *620*, 1089–1100.

[2] R. Krishna, J. Wang, W. Ahern, P. Sturmfels, I. Kalvet, G. R. Lee, F. S. Morey-Burrows, I. R. Humphreys, R. McHugh, D. Vafeados, X. Li, A. Sutherland, A. Hitchcock, C. N. Hunter, M. Baek, F. DiMaio, "Generalized Biomolecular Modeling and Design with RoseTTAFold All-Atom", *Science*, **2024**, Vol 384, Issue 6693.

[3] J. Butcher, R. Krishna, R. Mitra, R. I. Brent, Y. Li, N. Corley, P. Kim, J. Funk, S. Mathis, S. Salike, A. Muraishi, H. Eisenach, T. R. Thompson, J. Chen, Y. Politanska, E. Sehgal, B. Coventry, O. Zhang, B. Qiang, K. Didi, M. Kazman, F. DiMaio, D. Baker, "De novo design of all-atom biomolecular interactions with RFdiffusion3" *bioRxiv* **2025**, 2025.09.18.676967.

[4] H. Stark, F. Faltings, M. Choi, Y. Xie, E. Hur, T. O'Donnell, A. Bushuiev, T. Uçar, S. Passaro, W. Mao, M. Reveiz, R. Bushuiev, T. Pluskal, J. Sivic, K. Kreis, A. Vahdat, S. Ray, J. T. Goldstein, A. Savinov, J. A. Hambalek, A. Gupta, D. A. Taquiri-Diaz, Y. Zhang, A. K. Hatstat, A. Arada, N. H. Kim, E. Tackie-Yarboi, D. Boselli, L. Schnaider, C. C. Liu, G.-W. Li, D. Hnisz, D. M. Sabatini, W. F. DeGrado, J. Wohlwend, G. Corso, R. Barzilay, T. Jaakkola, **2025**, DOI: 10.1101/2025.11.20.689494.

[5] J. Dauparas, I. Anishchenko, N. Bennett, H. Bai, R. J. Ragotte, L. F. Milles, B. I. M. Wicky, A. Courbet, R. J. De Haas, N. Bethel, P. J. Y. Leung, T. F. Huddy, S. Pellock, D. Tischer, F. Chan, B. Koepnick, H. Nguyen, A. Kang, B. Sankaran, A. K. Bera, N. P. King, D. Baker, "Robust deep learning–based protein sequence design using ProteinMPNN" *Science* **2022**, *378*, 49–56.

[6] J. Dauparas, G. R. Lee, R. Pecoraro, L. An, I. Anishchenko, C. Glasscock, D. Baker, "Atomic context-conditioned protein sequence design using LigandMPNN" *Nat. Methods* **2025**, *22*, 717–723.

[7] A. Alexaki, J. Kames, D. D. Holcomb, J. Athey, L. V. Santana-Quintero, P. V. N. Lam, N. Hamasaki-Katagiri, E. Osipova, V. Simonyan, H. Bar, A. A. Komar, C. Kimchi-Sarfaty, "Codon and Codon-Pair Usage Tables (CoCoPUTs): Facilitating Genetic Variation Analyses and Recombinant Gene Design" *J. Mol. Biol.* **2019**, *431*, 2434–2441.

[8] H. M. Salis, E. A. Mirsky, C. A. Voigt, "Automated design of synthetic ribosome binding sites to control protein expression" *Nat. Biotechnol.* **2009**, *27*, 946–950.

[9] J. Jumper, R. Evans, A. Pritzel, T. Green, M. Figurnov, O. Ronneberger, K. Tunyasuvunakool, R. Bates, A. Žídek, A. Potapenko, A. Bridgland, C. Meyer, S. A. A. Kohl, A. J. Ballard, A. Cowie, B. Romera-Paredes, S. Nikolov, R. Jain, J. Adler, T. Back, S. Petersen, D. Reiman, E. Clancy, M. Zielinski, M. Steinegger, M. Pacholska, T. Berghammer, S. Bodenstein, D. Silver, O. Vinyals, A. W. Senior, K. Kavukcuoglu, P. Kohli, D. Hassabis, "Highly accurate protein structure prediction with AlphaFold" *Nature* **2021**, *596*, 583–589.

[10] S. Passaro, G. Corso, J. Wohlwend, M. Reveiz, S. Thaler, V. R. Somnath, N. Getz, T. Portnoi, J. Roy, H. Stark, D. Kwabi-Addo, D. Beaini, T. Jaakkola, R. Barzilay, "Boltz-2: Towards accurate and efficient binding affinity prediction" *bioRxiv* **2025**, 2025.06.14.659707.

[11] A. T. McNutt, P. Francoeur, R. Aggarwal, T. Masuda, R. Meli, M. Ragoza, J. Sunseri, D. R. Koes, "GNINA 1.0: molecular docking with deep learning" *J. Cheminformatics* **2021**, *13*, 43.

[12] M. Buttenschoen, G. M. Morris, C. M. Deane, "PoseBusters: AI-based docking methods fail to generate physically valid poses or generalise to novel sequences" *Chem. Sci.* **2024**, *15*, 3130–3139.

[13] A. Kuriata, A. M. Gierut, T. Oleniecki, M. P. Ciemny, A. Kolinski, M. Kurcinski, S. Kmiecik, "CABS-flex 2.0: a web server for fast simulations of flexibility of protein structures" *Nucleic Acids Res.* **2018**, *46*, W338–W343.

[14] M. Steinegger, J. Söding, "MMseqs2 enables sensitive protein sequence searching for the analysis of massive data sets" *Nat. Biotechnol.* **2017**, *35*, 1026–1028.

Appendix A – using and extending BioPipelines with coding agents

This appendix documents two end-to-end uses of a state-of-the-art coding agent (Claude Code, Opus 4.7) on the BioPipelines repository, using prompt files present in the ‘llm/’: ‘pipelines’ for writing workflows, and ‘development’. We also report the automatic testing of the new tool (ADMET-AI) on a SLURM HPC (via ssh) and on Google Colab (via official MCP). Highlighted in orange is input from the user, and in light and dark blue the questions and output of the assistant, respectively, and we add commentary in black.

Part 1 – Writing workflows

❯ follow llm/pipelines.md

● I'll read the file to follow its instructions.

Read 3 files (ctrl+o to expand)

● Context loaded. Ready when you have a task.

❯ Write a workflow to find candidate protein targets for urolithin A.

● I'll ask a few questions to scope this — urolithin A binding to "candidate proteins" can mean several different things.

● User answered Claude's questions:

· How should candidate proteins be sourced? → RCSB structural search

· What's the screening method? → Boltz2 cofolding (Recommended)

· Roughly how many candidates? → Whichever are found

· Cluster or Colab? → Both

● A few follow-ups since the candidate list is RCSB-driven and the scale isn't pre-set:

● User answered Claude's questions:

· What RCSB query should drive the candidate set? → Text/keyword search

· Cap on candidate count after RCSB hit dedup? → Cap at ~50

· Run plan → Cluster .py + Colab .ipynb

● I need the actual query string and a couple of execution details before writing.

● User answered Claude's questions:

· What's the RCSB query string? → Similarity search

· Should I check for an existing RCSB tool wrapper first? → Yes, use the tool

· How should UroA enter the pipeline? → Ligand by name (Recommended)

● Got it — similarity search means: use the RCSB tool to find structures with ligands chemically similar to UroA, then cofold UroA against each. Let me check the RCSB tool's exact API and then load task tools.

Read 1 file (ctrl+o to expand)

● Got it. Let me also check Boltz2's API and one example pipeline to ground the structure.

Searched for 5 patterns, read 4 files (ctrl+o to expand)

● I have what I need. Now let me set up tasks and write both pipelines. Key design decisions:

- RCSB query: RCSB.Chemical(uroA_smiles, match_type="fingerprint-similarity") to find structures with chemically similar bound ligands. Cap at 50.
- Ligand: Ligand("urolithin A", codes="URO").
- Cofolding: Boltz2(proteins=Each(rcsb_hits), ligands=Bundle(uroA)) so each structure × UroA produces one complex with affinity. Use Bundle on the (single) ligand to keep it as one entity per protein, and Each is the default anyway — actually with one ligand Each is fine.
- Ranking: Panda merge confidence + affinity + search_results (for organism/title), sort by affinity_pred_value, plot.
- Cluster sweep does all 50; Colab demo does top 5 via max_results=5.

Listed 1 directory (ctrl+o to expand)

● Write(my_pipelines\uroa_target_search.py)

⎿ Wrote 69 lines to my_pipelines\uroa_target_search.py

● Write(my_pipelines\uroa_target_search.ipynb)

⎿ Wrote 160 lines to my_pipelines\uroa_target_search.ipynb

● Wrote two pipelines under my_pipelines/ (gitignored — scp the .py to the cluster, open the .ipynb in Colab):

- uroa_target_search.py (cluster, 50 candidates):
  - RCSB(RCSB.Chemical(UROA_SMILES, match_type="fingerprint-similarity"), ...protein-only filter, max_results=50) — pulls structures with bound ligands chemically similar to UroA.
  - Boltz2(proteins=candidates, ligands=Ligand(smiles=UROA_SMILES, ...)) — cofolds UroA against every hit; affinity=True is the default.
  - Panda.merge of search_results + confidence + affinity → ranked CSV with organism / title alongside ipTM / pLDDT / affinity_pred_value.
  - Two plots: affinity vs ipTM scatter, and per-PDB binding-probability bars.
  - Resources: gpu="any", 24 h — Boltz2 on 50 small/medium proteins should fit comfortably.
- uroa_target_search.ipynb (Colab demo, 5 candidates): same logic, with the canonical setup cell + Boltz2.install(), and a final download() cell since /content/ is ephemeral.

A few things worth flagging before you run:
- I used the canonical UroA SMILES Oc1ccc2c(c1)oc(=O)c1cc(O)ccc12. Swap if you want a different tautomer.
- RCSB.Chemical similarity will likely return mostly bacterial / fungal proteins (the literature on urolithin / ellagic acid co-crystals is small). If you'd rather restrict to human, add StructureAttribute.PolymerMolecularFeatures.ScientificName.equals("Homo sapiens") — say the word and I'll add it.
- I did not pass MSAs; Boltz2 will fetch them via the public MMseqs2 server per protein. For 50 distinct proteins that's ~50 server queries — fine but not free.
Submit with ./submit my_pipelines/uroa_target_search.py once scp'd to the cluster.

The prompt file pipeline.md instructs the assistant to conduct a structured interview to better define the scope of the task, with a few suggested answers based on the codebase. The generated notebook is presented hereafter. It was uploaded to Google Colab directly, and run using a A100 GPU.

<u>Urolithin A — candidate protein target search (Colab demo)</u>

*Small-scale version of `uroa_target_search.py`: pulls **5** RCSB structures whose bound ligands are chemically similar to UroA, then cofolds UroA against each with Boltz2.*
The cluster `.py` runs the full sweep (50 candidates). This notebook is the inline demo — runs in one Colab session on a single GPU.

1. Setup — clone repo, install BioPipelines and Boltz2

<u>CELL 1</u>

```
!git clone https://github.com/locbp-uzh/biopipelines
%cd biopipelines
!pip install -e ".[all]"
!wget -q https://github.com/mamba-org/micromamba-releases/releases/latest/download/micromamba-linux-64 -O /usr/local/bin/micromamba && chmod +x /usr/local/bin/micromamba
!micromamba create -f environments/biopipelines.yaml -y
```

<u>CELL 2</u>

```
from biopipelines.pipeline import *
from biopipelines.boltz2 import Boltz2
with Pipeline("Setup", "install", description="Install Boltz2"):
```

```
Boltz2.install()
```

2. Pipeline

CELL 3

```
from biopipelines.pipeline import *
from biopipelines.entities import RCSB, Ligand
from biopipelines.rcsb import StructureAttribute, PolymerEntityType
from biopipelines.boltz2 import Boltz2
from biopipelines.panda import Panda
from biopipelines.plot import Plot

UROA_SMILES = "Oc1ccc2c(c1)oc(=O)c1cc(O)ccc12"

Pipeline(project="UrolithinA",
        job="TargetSearchDemo",
        description="Colab demo: cofold UroA against 5 RCSB hits")

uroa = Ligand(smiles=UROA_SMILES, ids="UroA", codes="URO")

candidates = RCSB(
    RCSB.Chemical(UROA_SMILES, match_type="fingerprint-similarity"),

StructureAttribute.PolymerMolecularFeatures.PolymerEntityType.equals(PolymerEntityType.PR
OTEIN),
    max_results=5,
    sort="score",
)
```

CELL 4

```
cofolded = Boltz2(proteins=candidates, ligands=uroa)
```

CELL 5

```
ranked = Panda(
    tables=[candidates.tables.search_results,
            cofolded.tables.confidence,
            cofolded.tables.affinity],
    operations=[
```

```
        Panda.merge(),
        Panda.calculate({"aff_uM": "10**affinity_pred_value"}),
        Panda.sort("affinity_pred_value", ascending=True),
    ],
)
Plot(
    Plot.Scatter(
        data=ranked.tables.result,
        x="iptm",
        y="aff_uM",
        color="affinity_probability_binary",
        title="UroA candidate targets — affinity vs interface confidence",
        xlabel="ipTM",
        ylabel="Predicted affinity [uM]",
    ),
    Plot.Bar(
        data=ranked.tables.result,
        x="pdb_id",
        y="affinity_probability_binary",
        title="UroA binding probability by PDB hit",
    ),
)
```

3. Save before the runtime ends

Outputs land in `/content/BioPipelines/UrolithinA/TargetSearchDemo_*/` and are wiped when the kernel disconnects. Mount Drive or download `ranked` / the scatter plot before closing.

CELL 6

```
ranked.download("./uroa_targets_ranked.csv")
```

All the cells run smoothly and produced the desired results, except the last cell. StandardizedOutput::download(self) doesn't accept an argument, instead it downloads the whole outputs. After removing the string argument, the ranked PDBs were successfully downloaded. We report here the resultings plots:

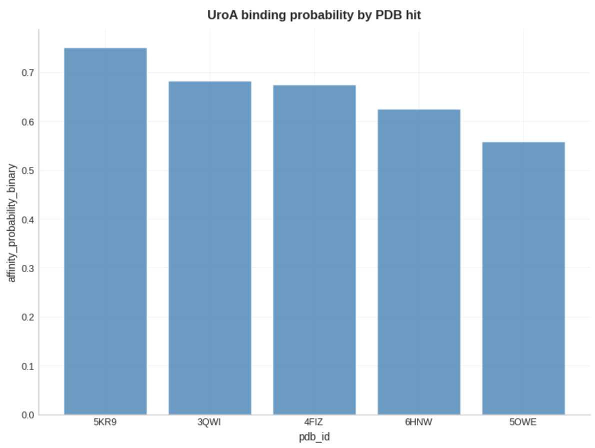

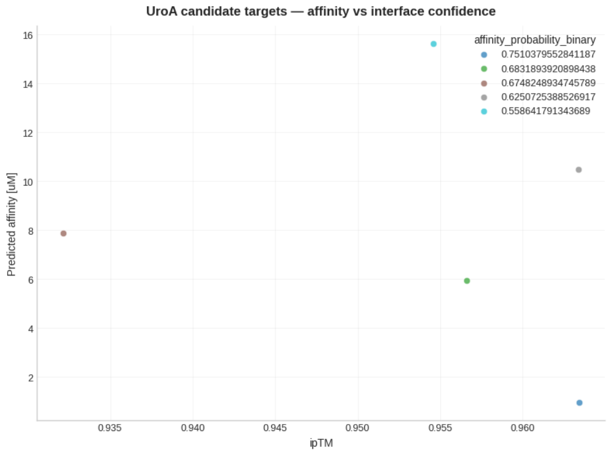

Part 2 – extending the framework

Tool development

❯ follow development.md

● I'll find and read development.md to follow its instructions.

Searched for 1 pattern, read 3 files (ctrl+o to expand)

● Context loaded. Ready for your task.

❯ implement ADMET-AI

● Before I touch anything, two questions to lock down branch and upstream reference.

● User answered Claude's questions:

· Where should the ADMET-AI work land? → New branch tool-admet-ai from main

· Is the upstream ADMET-AI the Swanson Lab repo at https://github.com/swansonk14/admet_ai, or a different one? → swansonk14/admet_ai

● Bash(git checkout -b tool-admet-ai)

⎿ Switched to a new branch 'tool-admet-ai'

● Now I'll review the upstream and existing compound-consuming tools to pick patterns per axis.

● Fetch(https://github.com/swansonk14/admet_ai)

⎿ Received 294.5KB (200 OK)

Read 1 file (ctrl+o to expand)

● Let me find a close compound-consuming analogue — PoseBusters processes compounds and produces only tables, similar to what ADMET-AI needs.

Searched for 2 patterns, read 1 file (ctrl+o to expand)

● Now let me check a tool that consumes the compounds stream — to see how SMILES

are read.

Searched for 2 patterns, read 1 file (ctrl+o to expand)

● Let me look at how Boltz2 handles compounds → SMILES extraction at runtime, and check pipe scripts for compound consumers.

Searched for 3 patterns, read 1 file (ctrl+o to expand)

● Now let me check the compounds-stream schema (does the SMILES live in value column of map_table or elsewhere?) and look at an existing pipe script that reads SMILES.

Searched for 3 patterns (ctrl+o to expand)

● Confirmed: the compounds stream's map_table has id, format, smiles, ccd columns. Now let me check pipe_smiles_properties.py to see how to read SMILES values from the stream at runtime.

Read 1 file (ctrl+o to expand)

● Let me check how that script gets invoked — does it consume the compounds map_table directly?

Searched for 1 pattern (ctrl+o to expand)

● Not actually wired up. Let me look at iterate_values from biopipelines_io for how to read SMILES from the compounds stream at runtime.

Searched for 1 pattern, read 1 file (ctrl+o to expand)

● Good. Now let me check how pipe_script_path works and see one or two existing tools' install patterns relevant for ADMET-AI (it is a pip package, not a separate repo — closer to PoseBusters).

Searched for 2 patterns, read 3 files (ctrl+o to expand)

● I have all the patterns I need. Let me also check __init__.py to know how to register the tool, the colab config, and tool_index.

Searched for 3 patterns, read 1 file (ctrl+o to expand)

● Now let me check the colab.yaml for pattern, the upstream admet_ai output format properly (need column names — let me fetch the README for the package):

● Fetch(https://raw.githubusercontent.com/swansonk14/admet_ai/main/README.md)

⎿ Received 5.4KB (200 OK)

Searched for 1 pattern (ctrl+o to expand)

● Python 3.14 sounds wrong (admet_ai 1.x supports older Python). Let me check the upstream pyproject directly.

● Fetch(https://raw.githubusercontent.com/swansonk14/admet_ai/main/pyproject.tom

⎿ Received 1.6KB (200 OK)

● Good — Python ≥3.11, the column structure isn't documented in README so I'll keep TableInfo columns minimal (just declare id and smiles as known and accept the rest as predictions written by the tool). Let me design and present the sketch now.

Here's my proposed design:
Tool: ADMET_AI (file: biopipelines/admet_ai.py, pipe: pipe_scripts/pipe_admet_ai.py)
- Inputs (per-axis pick):
  - compounds: Union[DataStream, StandardizedOutput] — the canonical compounds stream (analogue: PoseBusters' structures resolution; Boltz2's ligands_stream lookup). SMILES is read from the canonical smiles column at runtime via iterate_values(ds, columns=['smiles']).
- Configuration pattern: small JSON config written at config time (analogue: PoseBusters' posebusters_config.json), passed to the pipe script. Pipe script: read config → load compounds DataStream → build a temporary _configuration/admet_input.csv with id, smiles → call admet_predict --data_path ... --save_path ... --smiles_column smiles via subprocess → re-attach id column and write to tables/admet.csv.
- Execution pattern: single-shot CLI through pipe script (one batch over all SMILES — admet_predict is batch-friendly).
- Outputs:
  - Streams: none.
  - Tables: admet — id, smiles, <ADMET property columns…>. Since the upstream property column set isn't documented in README, I'll declare ["id", "smiles"] as known columns in TableInfo and note the remaining columns are appended at runtime (this matches the count field behavior — it's discovered post-execution).
- Env: dedicated env admet_ai (analogue: PoseBusters' posebusters env). Python 3.11, install admet-ai via pip. Files: environments/admet_ai.yaml + environments/admet_ai.pip.txt.
- ID handling: pass-through. Output IDs == input compound IDs.
- Parameters (single sketch, all implemented):
  - compounds (required) — DataStream/StandardizedOutput with compounds.

No other knobs. Notably deferred / not added:
  - No smiles_column= parameter — the framework pins the compounds-stream schema to the smiles column; we read that and pass --smiles_column smiles internally.
  - No GPU/device flag — admet_ai uses available hardware automatically.
- Config-vs-execution split: the wrapper class only writes the JSON config and emits bash that calls the pipe script. The pipe script is what reads the compounds map_table, constructs the

admet_predict input CSV, runs the binary, and joins the output back to IDs. Nothing in the wrapper touches data.

Does this match what you want? In particular: confirm that passing the whole compounds set as one batch (not per-ID iteration) is the right execution shape, and that no extra parameters (e.g. a subset of property columns, model selection) need to be exposed.

❯ yes go ahead

● Implementing now.

Read 1 file (ctrl+o to expand)

● Let me also check the pipe_script_path helper and how a tool currently gets installed via mamba env (paths in posebusters install include biopipelines from folders).

Searched for 1 pattern (ctrl+o to expand)

● Now let me also look quickly at how Plot/Distance (pure-table tools that produce only tables) declare empty streams:

Searched for 1 pattern (ctrl+o to expand)

● Right — PoseBusters' get_output_files returns only tables and output_folder, no DataStream.empty entries. I'll do the same. Now writing the files.

● Write(environments\admet_ai.yaml)

⎿ Wrote 6 lines to environments\admet_ai.yaml

● Write(environments\admet_ai.pip.txt)

⎿ Wrote 1 lines to environments\admet_ai.pip.txt

● Write(biopipelines\admet_ai.py)

⎿ Wrote 172 lines to biopipelines\admet_ai.py

● Write(pipe_scripts\pipe_admet_ai.py)

⎿ Wrote 95 lines to pipe_scripts\pipe_admet_ai.py

● Now register the tool in __init__.py and the env mappings in both configs.

● Update(biopipelines__init__.py)

⎿ Added 1 line

51 +from .admet_ai import ADMET_AI

● Update(biopipelines__init__.py)

⎿ Added 1 line

122 +   'ADMET_AI',

● Update(config.cluster.yaml)

⎿ Added 1 line

144 + ADMET_AI: "admet_ai"

● Update(config.colab.yaml)

⎿ Added 1 line

108 + ADMET_AI: "admet_ai"

● Now smoke-import to make sure the module loads cleanly.

● Bash(python -c "from biopipelines.admet_ai import ADMET_AI; from biopipelines import ADMET_AI as Top; print('OK', ADMET_AI is Top, ADMET_AI.TOOL_NAME)")

⎿ OK True ADMET_AI

● Smoke import passes. Per memory, I'm skipping local pytest (CI runs it on push) — this change is config-time wiring + a CLI-driven pipe script, which is the kind of thing CI's mock-based suite exercises only at the framework level.

What changed

- biopipelines/admet_ai.py — ADMET_AI tool, single compounds input, no other params. Writes a small JSON config and dispatches to the pipe script.

- pipe_scripts/pipe_admet_ai.py — reads (id, smiles) from the compounds map_table via iterate_values, calls admet_predict --data_path … --save_path …

--smiles_column smiles, joins predictions back to IDs, writes tables/admet.csv.

- environments/admet_ai.yaml + environments/admet_ai.pip.txt — Python 3.11 env, pip-installs admet-ai. Install hook does an import admet_ai verification before touching $INSTALL_SUCCESS.

- Registered in biopipelines/__init__.py (export + __all__) and mapped to env admet_ai in config.cluster.yaml and config.colab.yaml.

- Branch: tool-admet-ai (created from main).

Verifications I did / didn't run

- ✅ Smoke-import via python -c "from biopipelines.admet_ai import ADMET_AI" — module loads cleanly through the package.

- ❌ Did not run the install / GPU code path locally — neither admet-ai nor a GPU is available here. The import admet_ai verification gate in the install script is what surfaces a broken install at execution time.

- ❌ Did not run pytest locally (per your standing instruction; CI on push to tool-admet-ai is the test signal).

- ❌ Did not run a real end-to-end pipeline — needs a cluster or Colab run to confirm admet_predict's actual output column shape; the table is declared with ["id", "smiles"] known and the rest appended at runtime.

One caveat worth flagging

The README we have doesn't pin admet_predict's output column names, so I left the TableInfo.columns declaration at ["id", "smiles"] — the predicted-property columns are accreted at runtime. If you later want column references like admet.tables.admet.<property> to validate at config time, you'll need to enumerate the property set explicitly (or have the install step write them to a manifest the wrapper reads).

Automated debugging on a SLURM Cluster

❯ follow cluster.md to debug the tool. on the cluster. the repository lives at /home/gquarg/biopipelines

Read 1 file (ctrl+o to expand)

● Plan: push the branch, hard-reset the cluster checkout to it, install ADMET-AI, run a tiny pipeline against a small CompoundLibrary, tail the slurm log.

Before the destructive git reset --hard origin/tool-admet-ai on the cluster: confirm that's OK?

● User answered Claude's questions:

⎿ · OK to hard-reset /home/gquarg/biopipelines to origin/tool-admet-ai? Any uncommitted edits there will be discarded. → Yes, hard-reset

● Pushing the branch first.

● Bash(git status --short && git log --oneline -5)

⎿ M biopipelines/__init__.py

● Need to commit before pushing. I'll commit the ADMET-AI scaffolding.

● Bash(git add biopipelines/admet_ai.py biopipelines/__init__.py
pipe_scripts/pipe_admet_ai.py environments/admet_ai.yaml
environments/admet_ai.pip.txt config.cluster.…)

● Bash(git push -u origin tool-admet-ai)

● Bash(ssh cluster "cd /home/gquarg/biopipelines && git checkout tool-admet-ai && git reset --hard origin/tool-admet-ai && git log --oneline -2…)

● Now write a minimal test pipeline (a 2–3 compound library + ADMETAI), copy it, and submit.

● Write(my_pipelines\test_admetai.py)

⎿ Wrote 20 lines to my_pipelines\test_admetai.py

● Wait — let me confirm Resources is exposed where I think it is.

Searched for 3 patterns (ctrl+o to expand)

● Good — Resources lives in biopipelines.pipeline. Let me fix the import in my test pipeline and check an example pipeline as a reference.

Searched for 1 pattern, read 1 file, listed 1 directory (ctrl+o to expand)

● Idiomatic pattern is from biopipelines.pipeline import *. Let me fix the test pipeline.

● Write(my_pipelines\test_admetai.py)

⎿ Added 2 lines, removed 3 lines

3 -from biopipelines.pipeline import Pipeline

3 +from biopipelines.pipeline import *

6 -from biopipelines.base_config import Resources

● Bash(ssh cluster "mkdir -p /home/gquarg/biopipelines/my_pipelines")

● Bash(scp my_pipelines/test_admetai.py cluster:/home/gquarg/biopipelines/my_pipelines/)

● Now I need to install ADMETAI first, then run the prediction. The most efficient approach is one pipeline that does both: ADMETAI.install() followed by the ADMETAI(...) call. Let me check if mixing install + run in one pipeline is supported.

Searched for 1 pattern, read 1 file (ctrl+o to expand)

● Confirmed — mixing .install() with the actual tool call in one pipeline is the canonical pattern. Updating the test pipeline.

● Update(my_pipelines\test_admetai.py)

⎿ Added 2 lines

13 +   ADMETAI.install()

14 +

● Bash(scp my_pipelines/test_admetai.py cluster:/home/gquarg/biopipelines/my_pipelines/ && ssh cluster "cd /home/gquarg/biopipelines && ./submit my_pipelin…)

● Job 2772482 submitted. Note the run dir: /shares/locbp.chem.uzh/gquarg/BioPipelines/Tests /admetai_smoke_001/. Now let me poll status.

● Bash(ssh cluster "squeue -j 2772482 -o '%.18i %.9P %.20j %.8T %.10M %R' 2>/dev/null; echo --- ; tail -n 80 /shares/locbp.chem.uzh/gquarg/BioPipelines/Tests/admetai_s…)

● Good — env was built cleanly, and we have a clear new signature now:

ModuleNotFoundError: No module named 'pkg_resources' (not the old "configparser" message). The setuptools line in admet_ai.pip.txt doesn't actually fix it, because pip resolves dependencies first then installs, and the install set above shows setuptools was not in the final list — pip saw it was already in the env (a conda default), then later something stripped it (or chemprop's pinned environment markers removed it).

Let me confirm what's actually in the env right now before proposing a fix.

● Bash(ssh cluster "mamba run -n admet_ai python -c 'import sys; print(sys.version)'; mamba run -n admet_ai pip show setuptools 2>&1 | head -3; echo --- ; mamba run -n…)

⎿ Error: Exit code 1

bash: line 1: mamba: command not found

● Need the shell hook. Let me probe properly:

● Bash(ssh cluster "module load miniforge3 2>&1 >/dev/null; eval \"\$(mamba shell hook --shell bash)\"; mamba activate admet_ai && python -c 'import sys; print(sys.ver…)

⎿ Error: Exit code 127

bash: line 1: module: command not found

● Login shells need lmod sourced. Let me just call the env's python directly — it exists at a known path.

● Bash(ssh cluster "/home/gquarg/data/conda/envs/admet_ai/bin/python -c 'import sys; print(sys.version)'; /home/gquarg/data/conda/envs/admet_ai/bin/pip list 2>/dev/nul…)

⎿ 3.10.20 | packaged by conda-forge | (main, Mar 5 2026, 16:42:22) [GCC 14.3.0]

admet_ai 1.4.0

… +4 lines (ctrl+o to expand)

● setuptools 82.0.1 IS installed, but pkg_resources still fails to import. That's the real bug: setuptools 82+ no longer ships pkg_resources by default — it was split into the separate setuptools-pkg-resources / requires explicit opt-in. The pip.txt fix is wrong because the version of setuptools that gets installed is too new.

Let me confirm:

● Bash(ssh cluster "/home/gquarg/data/conda/envs/admet_ai/bin/python -c 'import
pkg_resources' 2>&1 | head -5; echo --- ;
/home/gquarg/data/conda/envs/admet_ai/bin/pyt…)

⎿ Traceback (most recent call last):

File "<string>", line 1, in <module>

ModuleNotFoundError: No module named 'pkg_resources'

… +9 lines (ctrl+o to expand)

● Confirmed — setuptools 82 has dropped pkg_resources.

Fix: pin setuptools<81 in admet_ai.pip.txt. Setuptools 80 still ships pkg_resources.

● Update(environments\admet_ai.pip.txt)

⎿ Added 3 lines, removed 1 line

2 -setuptools

2 +# setuptools<81 needed: chemprop 1.6.1 -> hyperopt 0.2.7 imports
  + pkg_resources,
3 +# which setuptools removed in 81+.
4 +setuptools<81

● Now I'll commit the fix, push, sync the cluster, and resubmit with force_reinstall=True.

● Bash(git add environments/admet_ai.pip.txt && git commit -m "ADMETAI: pin setuptools<81 so pkg_resources is shipped…)

● Bash(ssh cluster "cd /home/gquarg/biopipelines && git fetch && git reset--hard origin/tool-admet-ai && git log --oneline -2 && echo --- && cat enviro…)

● Bash(ssh cluster "cd /home/gquarg/biopipelines && ./submit my_pipelines/test_admetai.py 2>&1 | tail -15")

● Job 2772793 submitted to admetai_smoke_002. Force-reinstall again pulls torch + chemprop, ~3–4 min, then the predict step.

● Diagnosis: setuptools 81+ removed pkg_resources, but chemprop 1.6.1 → hyperopt 0.2.7 imports it unconditionally. The previous unpinned setuptools line in admet_ai.pip.txt resolved to 82.0.1 — too new. Pinned to setuptools<81, committed, pushed, synced, and resubmitted as job 2772793.

● Resuming check on job 2772793.

● Bash(ssh cluster "squeue -j 2772793 -o '%.18i %.9P %.20j %.8T %.10M %R' 2>/dev/null; echo --- ; sacct -j 2772793 --format=JobID,JobName,State,ExitCode,Elapsed -P 2>/…)

● Good — install in progress, the uninstall of setuptools 82.0.1 confirms the pin is taking effect. Job's been running 3:40, install nearly done. Let me wait a bit more.

● Job 2772793 still running, install reached the late stage (uninstalling old setuptools 82). Sleeping ~2 min for the rest of the install + verification + predict step.

● Bash(ssh cluster "squeue -j 2772793 -o '%.18i %.9P %.20j %.8T %.10M %R' 2>/dev/null; echo --- ; sacct -j 2772793 --format=JobID,JobName,State,ExitCode,Elapsed -P 2>/…)

● Job COMPLETED. All three steps passed: install, CompoundLibrary, ADMETAI. Let
  me grab the result CSV and verify shape.

● Bash(ssh cluster "head -1 /shares/locbp.chem.uzh/gquarg/BioPipelines/Tests/admetai_smoke_002/003_ADMETAI/tables/admet.csv | tr ',' '\n' | head -10; echo '...' ; head…)

● End-to-end pass. The output CSV at tables/admet.csv has:
  - 100 columns total (id, smiles, ~98 ADMET endpoints)
  - 3 rows, one per input compound, IDs preserved (aspirin, caffeine, ibuprofen)
  - Sensible numbers (e.g. aspirin MW 180.16, ibuprofen logP 3.07)

Summary

Bug: setuptools 81+ removed the bundled pkg_resources module, but chemprop 1.6.1 → hyperopt 0.2.7 imports it unconditionally. The previous unpinned setuptools line in admet_ai.pip.txt was resolving to 82.0.1, so ADMETModel() died at import.

Fix (a7f998c): pinned setuptools<81 in environments/admet_ai.pip.txt, with a comment explaining why.

Verification: job 2772793 (admetai_smoke_002) ran the full chain — force reinstall → CompoundLibrary → ADMETAI — to completion in 4:07. Output admet.csv is shaped correctly (3 rows × 100 cols). The branch tool-admet-ai is now in a working state on origin.

With minimal input from the user, the tool was successfully implemented, and a bug in the installation was automatically fixed. The fact that the tools accept standardized inputs ensures that after testing the tool with a simple input library of three compounds, it can now be chained to tool producing a compounds stream. Hereafter we report the first columns of the produced admet.csv table:

id	smiles	molecular_weight	logP	hydrogen_bond_acceptors
**aspirin**	CC(=O)OC1=CC=CC=C1C(=O)O	180.159	1.3101	3
**caffeine**	CN1C=NC2=C1C(=O)N(C(=O)N2C)C	194.194	-1.0293	3
**ibuprofen**	CC(C)CC1=CC=C(C=C1)C(C)C(=O)O	206.285	3.0732	1

Automated debugging on Google Colab

❯ now follow llm/colab.md.

Read 1 file (ctrl+o to expand)

● I need to load the colab-mcp tools first (open_colab_browser_connection unlocks the rest). Let me get its schema.

● Plan: open colab connection, then walk through the canonical 4-cell sequence — clone gianluca-main, set up biopipelines + micromamba, run ADMETAI.install(), and run a minimal ADMETAI pipeline. I'll let you pick the runtime (T4 is enough — no GPU needed for ADMETAI).

Called colab-mcp (ctrl+o to expand)

● Connected. Adding cell 1 — clone the gitlab repo on gianluca-main. The repo lives at gitlab.uzh.ch/locbp/public/biopipelines-locbp. Let me try the public path first.

Called colab-mcp 3 times (ctrl+o to expand)

● could not read Username for 'https://gitlab.uzh.ch', the repo requires auth (UZH-internal "public" ≠ anonymous). I need a token. Per colab.md, I should prompt the user for one interactively in the notebook so the value doesn't go through transcripts/MCP logs.

Called colab-mcp (ctrl+o to expand)

● Need a GitLab token for the clone. Per colab.md: create a project access token on gitlab.uzh.ch/locbp/public/biopipelines-locbp with role Reporter and scope read_repository. Then run the cell — getpass will prompt for it inside Colab so the value isn't logged here. Updating cell 1 to ask for it interactively.

Called colab-mcp (ctrl+o to expand)

● Cell kEstGPsqi2pt is ready. Since this cell uses input()/getpass(), it needs you to type in the Colab UI when it runs — the MCP run_code_cell call will block on those prompts.

Could you:

1. Create a project access token on the GitLab project (gitlab.uzh.ch/locbp/public/biopipelines-locbp → Settings → Access Tokens → role Reporter, scope read_repository).

2. Open the Colab notebook and run cell 1, entering the token name and value at the prompts.

Once the clone succeeds, tell me and I'll add the remaining cells (BioPipelines install + micromamba env, ADMETAI.install(), the minimal pipeline).

❯ done

● Checking that the clone landed.

Called colab-mcp (ctrl+o to expand)

● Clone succeeded; CWD is /content/biopipelines. Now adding cell 2 — install BioPipelines + micromamba — per the canonical Colab setup.

Called colab-mcp 2 times (ctrl+o to expand)

● Output went to a file. Let me grab the tail (success/failure markers).

Read 1 file (ctrl+o to expand)

● Cell 2 finished cleanly — biopipelines env created via micromamba. Now cell 3: ADMETAI.install().

Called colab-mcp 2 times (ctrl+o to expand)

● Install completed (setuptools downgraded to 80.10.2, all deps installed), but the verification step says "cannot import admet_ai or load model". That's the install script's from admet_ai import ADMETModel; ADMETModel() test. Need to see the actual error from that import — the current verify line swallows stderr (>/dev/null 2>&1).

Let me probe directly.

Called colab-mcp 2 times (ctrl+o to expand)

● Real culprit, totally distinct from cluster: Colab leaks MPLBACKEND=module://matplotlib_inline.backend_inline into the child env's environment. The fresh admet_ai env doesn't have matplotlib_inline installed, so matplotlib's import refuses the backend and crashes — which chemfunc → admet_ai trips on.

Two options:

- A. Strip MPLBACKEND in the install script's verify block (and ideally also when the tool actually runs at execution time). This is a Colab-specific bug, but fixing it in the install script makes the tool robust to inherited matplotlib state from notebook hosts.

- B. Set MPLBACKEND=agg before the verify — a cleaner per-call override. Same idea, narrower scope.

I'll go with B inside activate_environment consumer paths plus the install verify, since the same env-leak will hit the pipe script at run time too. Actually — let me first prove the runtime hit. The pipe script imports admet_ai under the same conditions.

Let me confirm and pick the cleanest fix. First, check whether MPLBACKEND is set as a process env or just as an rcParams value at notebook-runtime.

Called colab-mcp 2 times (ctrl+o to expand)

● Diagnosis fully confirmed:

1. os.environ['MPLBACKEND'] is 'module://matplotlib_inline.backend_inline' — set in every Colab process, inherited by micromamba run child.

2. With MPLBACKEND=agg prefix, ADMETModel() instantiates cleanly. So the fix is one line.

Now: this is a problem at execution time (the pipe script also runs from admet_ai import ADMETModel), not just install verify. The most robust fix is to neutralize the offending value inside pipe_admet_ai.py itself before importing admet_ai. That covers both the install-verify path and every actual pipeline run. (Setting it in the install verify alone wouldn't help the predict step.)

Let me apply it:

Read 1 file (ctrl+o to expand)

● Update(pipe_scripts\pipe_admet_ai.py)

⎿ Added 7 lines

● Now also need to fix the install-verify, because right now ADMETAI.install() is reporting FAILED even though the env is fine — so the framework will keep re-running install / refusing to proceed.

Read 1 file (ctrl+o to expand)

● Update(biopipelines\admet_ai.py)

⎿ Added 1 line, removed 1 line

● Commit and push so a fresh clone gets the fix:

● Bash(git add biopipelines/admet_ai.py pipe_scripts/pipe_admet_ai.py && git commit -m "ADMETAI: override MPLBACKEND=agg before importing admet_ai…)

⎿ [gianluca-main 1de3837] ADMETAI: override MPLBACKEND=agg before importing admet_ai

2 files changed, 8 insertions(+), 1 deletion(-)

● Now I'll have the notebook pull the new commit (no need to reclone — git pull is enough), retouch the install marker so the next .install() call sees the env as good, and run the actual ADMETAI pipeline.

Two options for the marker:

- Re-run ADMETAI.install() — it'll re-check the skip path (env exists), now touch SUCCESS, exit 0.

- Or run force_reinstall=True — wastes ~3 minutes downloading torch again.

The skip path uses _env_exists_check, which only checks the env is present. So ADMETAI.install() (no force) will just touch $INSTALL_SUCCESS and return. Let me add the pull cell + a fresh install-check cell + the pipeline.

Called colab-mcp 2 times (ctrl+o to expand)

● Install marker refreshed (ADMETAI_installation_COMPLETED). Pull also picked up unrelated changes (cabsflex.py, protein_mpnn.py) — interesting since gianluca-main was supposed to be the squashed merge of everything, but no matter, they don't affect us.

Now the actual pipeline:

Called colab-mcp 2 times (ctrl+o to expand)

● End-to-end pass on Colab. ADMETAI ran on 3 compounds in seconds, produced admet.csv with shape (3, 100) — same column count as the cluster run. Values look sensible:

id	MW	logP	QED
**aspirin**	180.16	1.31	0.55
**caffeine**	194.19	-1.03	0.54
**ibuprofen**	206.29	3.07	0.82

Summary

Bug on Colab: Colab leaks MPLBACKEND=module://matplotlib_inline.backend_inline into every subprocess. The dedicated admet_ai micromamba env doesn't have the matplotlib_inline shim, so the transitive import admet_ai → chemfunc → matplotlib.pyplot crashed with 'is not a valid value for backend'. This was a pure host-env-leak bug, completely separate from the cluster's setuptools<81 issue.

Fix (commit 1de3837): force MPLBACKEND=agg in two places:

- pipe_scripts/pipe_admet_ai.py — at the top of the file, before any imports that pull matplotlib (so the predict step works under any host that leaks an exotic MPLBACKEND).
- biopipelines/admet_ai.py install-verify line — so .install() doesn't spuriously mark the env FAILED on Colab.

Verification on Colab: ADMETAI.install() returns COMPLETED, the 3-compound pipeline runs cleanly, output table has the expected 100 columns and 3 rows with chemically-plausible values. Both Colab and cluster verifications now pass.

Again, with minimal input from the user, the tool was successfully implemented in Colab as well. The coding assistat run into a Colab-specific bug that affected the import, namely, an incompatible backend for MatPlotLib. The bug was automatically solved.

Appendix B – BioPipelines vs manual scripting

For each of the five case-study workflows of the main text, we compare the BioPipelines implementation, as found in the public github repository, with the equivalent built as hand-written cluster scripts. For BioPipelines we report the file and its size in non-blank, non-comment lines. For the manual version we list the artifacts a user would actually have to write as code, separately from the steps a user would typically just do by hand interactively. We also note that inter-tool file-format conversions, residue-set bookkeeping, control flow, and scheduler directives are absorbed by BioPipelines into the framework.

Application 1 (ubiquitin.py – 6 tools, 25 lines)
Redesign ubiquitin (PDB 4LCD, chain E) with the soluble ProteinMPNN model (10 sequences); fold all with AlphaFold2; compute the Cα-RMSD of each to the parent; keep sequences with RMSD < 1.5 and pLDDT > 80; codon-optimize the survivors for *E. coli*. Six tool calls; no user-written glue, no format conversions, no scheduler directives.

Manual scripting

Artifact	Responsibility
01_proteinmpnn.sbatch	#SBATCH headers + module load/mamba activate; run protein_mpnn_run.py with the soluble weights, --num_seq_per_target 10; collect the seqs/ FASTA
make_af2_fasta.py	strip the native record from each ProteinMPNN file; concatenate the 10 designs into one AF2 input FASTA with unique headers; record the design→sequence map
02_alphafold.sbatch	headers + env (localColabFold has no conda env — call the binary by path); colabfold_batch
rmsd_filter.py	parse each rank_001 score JSON for pLDDT; superpose each model on 4LCD chain E and compute Cα-RMSD; build a table; keep RMSD < 1.5 and pLDDT > 80
codon_optimize.py	codon-optimize each surviving sequence for *E. coli* (organism codon-usage table; rare-codon avoidance / repeat reduction) → DNA FASTA

User: fetch 4LCD, extract chain E, clean it, transfer to the cluster; submit the stages.

Application 2 (kinase_LID_redesign.py – 8 tools, 31 lines)
Replace the LID domain of adenylate kinase (PDB 4AKE) with a 50–70-residue backbone (RFdiffusion, 10 designs); 2 ProteinMPNN sequences per backbone, redesigning only the per-

backbone diffused segment; AlphaFold2 on all; keep structures whose non-designed core matches the original (Cα-RMSD < 1.5); rank by pLDDT; render the top 3 in a PyMOL session colored by AF2 confidence.

Manual scripting:

Artifact	Responsibility
01_rfdiffusion.sbatch	headers + env; run_inference.py inference.input_pdb=4AKE.pdb 'contigmap.contigs=[A1-117/50-70/A161-214]' inference.num_designs=10 …
parse_trb.py	per design_*.trb: read the contig record; compute the diffused-residue and fixed-core indices in the renumbered output PDB; write a per-design fixed_positions.jsonl + chain-list file in ProteinMPNN's expected format
02_proteinmpnn.sbatch	headers + env; loop over the 10 backbones, protein_mpnn_run.py --pdb_path … --fixed_positions_jsonl … --num_seq_per_target 2
collect_fastas.py	walk ProteinMPNN's seqs/, drop native records, concatenate the 20 sequences into one AF2 FASTA, record the design→sequence map
03_alphafold.sbatch	headers + env; colabfold_batch --msa-mode single_sequence …
rmsd_and_rank.py	parse AF2 score JSONs for pLDDT; superpose each model on 4AKE over the fixed-core Cα atoms (using the residue map from parse_trb.py); compute Cα-RMSD; filter RMSD < 1.5, sort by pLDDT, take top 3

User: fetch and clean 4AKE; load the ranked top-3 PDBs and make the colored PyMOL session; submit the stages.

Application 3 (compound_screening.py – 5 tools, 32 lines)

Screen a combinatorial library of tryptophan derivatives (built from a ChemDraw file with R-group tables) against the tryptophan-repressor homodimer in the presence of its DNA operator (PDB 1TRO); rank by predicted binding affinity; plot affinity vs. the R1 substituent.

Manual scripting:

Artifact	Responsibility
build_library.py	parse the ChemDraw .cdxml + R-group tables; enumerate the combinatorial library; emit a SMILES (and CCD/SDF where needed) per compound and a table recording each compound's R-group identities
make_boltz_inputs.py	per compound, write a Boltz-2 input YAML with two TrpR chains + the dsDNA + that ligand (correct chain types, MSA handling, ligand as SMILES/CCD) — one job per compound
01_boltz_array.sbatch	a SLURM array job over the per-compound YAMLs; headers + mamba activate Boltz2Env; boltz predict …
gather_affinities.py	parse each affinity_*.json; pull affinity_pred_value / affinity_probability_binary; join with the R-group table on compound id; compute aff_uM = 10**affinity_pred_value

<u>User:</u> draw and curate the ChemDraw R-group library; make the affinity-vs-R1 figure; submit the stages (sbatch --dependency=afterok: or a small driver).

Application 4 (FRET.py – 15 tools, 69 lines)

Build a calmodulin-based FRET sensor by fusing EBFP–CaM–EYFP with variable-length flexible linkers (4×4 = 16 constructs); predict each in apo and Ca^{2+}-bound forms with Boltz-2 (recycling the apo MSA into the holo run); measure the donor–acceptor chromophore distance in both states; derive FRET efficiency and its calcium-dependent change; plot per linker combination; render the best construct in PyMOL.

<u>Manual scripting:</u>

Artifact	Responsibility
build_fusions.py	enumerate the 4×4 linker-length combinations; build the 16 fused sequences (donor–linker–CaM–linker–acceptor); record each construct's domain boundary residue ranges
make_boltz_inputs.py	per construct, write the apo Boltz YAML; and the holo YAML with four Ca^{2+} ions, pointing at the apo run's MSA so it is not recomputed; manage the apo→holo dependency per construct
01_boltz_apo.sbatch<br>02_boltz_holo.sbatch	SLURM array jobs over the constructs (holo depends on apo per construct); headers + mamba activate Boltz2Env; boltz predict …
chromophore_distance.py	for each apo and holo model, locate the donor chromophore residue (66) and the acceptor chromophore residue (third from

Artifact	Responsibility
	the C-terminus); measure their distance; build apo/holo distance tables
fret_metrics.py	join the distance tables with the construct table; compute FRET_E_apo, FRET_E_holo, delta_FRET (Förster equation, R0 = 35.4 Å); sort by delta_FRET

<u>User:</u> make the two bar plots; for the best construct, make a PyMOL session coloring each of the three domains; submit the stages (sbatch --dependency=afterok: or a small driver).

Application 5 (iterative_binding_optimization.py – 4 + 6 x N tools, 40 lines)
Co-fold the wild-type protein–ligand complex with Boltz-2 for a reference affinity, then 5 cycles: pocket = residues within 5 Å of the ligand in the current best structure (recomputed each cycle); 1000 LigandMPNN sequences redesigning only the pocket; build a per-position mutation-frequency profile vs. the current best; compose 3 mutants by weighted-random sampling (≤ 3 substitutions each); co-fold them with Boltz-2; keep the highest affinity_probability_binary (vs. the previous best) as the next seed.
<u>Manual scripting:</u>

Artifact	Responsibility
00_reference_boltz.sbatch	headers + mamba activate Boltz2Env; write the Boltz-2 YAML for the WT protein + ligand (SMILES/CCD); boltz predict …; parse the affinity JSON; designate the seed structure; write a small state file
pocket_residues.py	load the current-best structure; find all protein residues with any atom within 5 Å of any ligand atom; write the pocket list and its complement in LigandMPNN's --fixed_residues format
01_ligandmpnn.sbatch	headers + mamba activate ligandmpnn_env; LigandMPNN/run.py --model_type ligand_mpnn --pdb_path … --fixed_residues "$(cat …)" --number_of_… 1000 … (correct model-params path)
mutation_profile.py	read the 1000 designed FASTA records; align each to the current-best sequence; tally per-position substitution counts → frequency table CSV

Artifact	Responsibility
compose_mutants.py	from the frequency table, sample 3 mutant sequences by weighted-random choice of substitutions, enforcing ≤ 3 mutations per mutant → 3 FASTA records
make_boltz_inputs.py	for each of the 3 mutants, write a Boltz-2 YAML with that sequence + the ligand, reusing the reference run's cached MSA
02_boltz_candidates.sbatch	headers + env; boltz predict on the 3 YAMLs
pick_best.py	parse the 3 affinity JSONs; compare affinity_probability_binary against the previous best held in the state file; write the new best (sequence + structure path) back to the state file
finalize.py	after 5 cycles: report the overall best sequence, its affinity_probability_binary, and its Boltz-2 structure path
run_cycles.sh	drive cycles 0→5: each cycle's submission depends on the previous cycle's *result* (the carried-over best), not just job completion — so unlike the linear pipelines this cannot be done by hand at the terminal; thread the state file through --dependency=afterok: chains, handle job IDs and failures across cycles

User: loop, state carry-over, cross-cycle dependency wiring.
